# Depth- and range-dependent variation in the performance of aquatic telemetry systems: understanding and predicting the susceptibility of acoustic tag–receiver pairs to close proximity detection interference

**DOI:** 10.7717/peerj.4249

**Published:** 2018-01-12

**Authors:** Stephen R. Scherrer, Brendan P. Rideout, Giacomo Giorli, Eva-Marie Nosal, Kevin C. Weng

**Affiliations:** 1 Department of Oceanography, University of Hawaiʻi at Mānoa, Honolulu, HI, USA; 2 Department of Ocean and Resources Engineering, University of Hawaiʻi at Mānoa, Honolulu, HI, USA; 3 National Institute of Water and Atmospheric Research Ltd., Wellington, New Zealand; 4 Virginia Institute of Marine Science, College of William and Mary, Gloucester Point, VA, USA

**Keywords:** Doughnut effect, Acoustic telemetry, CPDI, Prediction model, Multipath, Close proximity detection interference, Range test, Vemco

## Abstract

**Background:**

Passive acoustic telemetry using coded transmitter tags and stationary receivers is a popular method for tracking movements of aquatic animals. Understanding the performance of these systems is important in array design and in analysis. Close proximity detection interference (CPDI) is a condition where receivers fail to reliably detect tag transmissions. CPDI generally occurs when the tag and receiver are near one another in acoustically reverberant settings. Here we confirm transmission multipaths reflected off the environment arriving at a receiver with sufficient delay relative to the direct signal cause CPDI. We propose a ray-propagation based model to estimate the arrival of energy via multipaths to predict CPDI occurrence, and we show how deeper deployments are particularly susceptible.

**Methods:**

A series of experiments were designed to develop and validate our model. Deep (300 m) and shallow (25 m) ranging experiments were conducted using Vemco V13 acoustic tags and VR2-W receivers. Probabilistic modeling of hourly detections was used to estimate the average distance a tag could be detected. A mechanistic model for predicting the arrival time of multipaths was developed using parameters from these experiments to calculate the direct and multipath path lengths. This model was retroactively applied to the previous ranging experiments to validate CPDI observations. Two additional experiments were designed to validate predictions of CPDI with respect to combinations of deployment depth and distance. Playback of recorded tags in a tank environment was used to confirm multipaths arriving after the receiver’s blanking interval cause CPDI effects.

**Results:**

Analysis of empirical data estimated the average maximum detection radius (AMDR), the farthest distance at which 95% of tag transmissions went undetected by receivers, was between 840 and 846 m for the deep ranging experiment across all factor permutations. From these results, CPDI was estimated within a 276.5 m radius of the receiver. These empirical estimations were consistent with mechanistic model predictions. CPDI affected detection at distances closer than 259–326 m from receivers. AMDR determined from the shallow ranging experiment was between 278 and 290 m with CPDI neither predicted nor observed. Results of validation experiments were consistent with mechanistic model predictions. Finally, we were able to predict detection/nondetection with 95.7% accuracy using the mechanistic model’s criterion when simulating transmissions with and without multipaths.

**Discussion:**

Close proximity detection interference results from combinations of depth and distance that produce reflected signals arriving after a receiver’s blanking interval has ended. Deployment scenarios resulting in CPDI can be predicted with the proposed mechanistic model. For deeper deployments, sea-surface reflections can produce CPDI conditions, resulting in transmission rejection, regardless of the reflective properties of the seafloor.

## Introduction

The past three decades have seen an increase in the popularity of passive tracking of aquatic animals using acoustic telemetry systems (Heupel & Webber, 2012). Due in part to the relatively low cost to acquire large amounts of data, adaptability to a range of taxa, and ease of use by a global community of researchers, these systems are useful for answering a host of ecological questions including those concerning spatial use and management, home range size, migratory behaviors, and mortality rates (Heupel & Webber, 2012; Kessel et al., 2015). Established in 1979, Vemco Ltd. is the market-leading manufacturer of aquatic passive acoustic tracking systems (VEMCO, 2015). Their systems consist of two primary components; a transmitter tag attached to the study organism and a stationary receiver unit which detects coded acoustic transmissions from the tag, indicating the presence of a tagged individual in the detection region of the receiver.

Interpretation of the results of a telemetry study requires knowledge of the receiver’s detection region to understand the probability of a transmission’s detection across a range of potential depths and distances which a tagged individual may occupy. The passive sonar equation provides a framework for understanding factors affecting detection of transmissions.

$${\rm{SL}} - {\rm{TL}} - {\rm{NL}} > {\rm{DT}}$$

A transmission is likely to be detected when the signal-to-noise ratio of the arriving ping exceeds the receiver’s detection threshold (DT). The received level (RL) depends on the source level (SL) and transmission loss (TL), including geometric spreading and attenuation via scattering and absorption. A signal can be detected when the RL exceeds the background noise level (NL) by a level greater than the DT in the frequency range of interest (Urick, 1967). The NL of an environment fluctuates over time, with abiotic, biotic, and anthropogenic sources contributing to environmental background noise. Abiotic sources affecting passive acoustic telemetry systems include ocean tides and waves, stratification, weather events, and the absorptive and reflective acoustical properties of the environment. Sources of biotic noise include snapping shrimp, mantis shrimp, urchins, some reef fish, and cetaceans (Cagua, Berumen & Tyler, 2013; Gjelland & Hedger, 2013; Kessel et al., 2013; Mathies et al., 2014). For a given signal level, detection probability is generally improved in cases with lower TL and lower NLs.

Propagation conditions, TLs, and NLs differ across sites; therefore determining the detection characteristics of receivers for every study is critical. A 2013 meta-analysis of 321 acoustic tracking studies called for more comprehensive detection range testing and reporting in acoustic tagging studies, finding that only 48.6% of studies reviewed included results from equipment ranging experiments (Kessel et al., 2013). Some of the ways a receiver’s effective detection range has been determined include citing previously published studies (Kessel et al., 2013), modeling the effects of environmental parameters based on the study site using tools provided by the manufacturer (Parrish et al., 2015), and empirical range testing involving measurement of tag detections at receivers in conditions similar to the proposed study site (Simpfendorfer, Heupel & Collins, 2008).

A common finding of range testing experiments is that the probability of detecting a transmission decreases with increasing range between a tag and receiver, with the highest probability of detection occurring when tags are at distances closest to the receiver (Simpfendorfer, Heupel & Collins, 2008). However, under some circumstances, detection probabilities for tags in close proximity to the receiver unit can be low, with the peak probability of detection occurring at some intermediate distance from the receiver unit. Kessel et al. (2015) termed this phenomenon “close proximity detection interference,” CPDI. The study identified acoustically reflective environments with strong echoes as particularly susceptible to these effects.

Observations of CPDI have been noted in other acoustic ranging experiments (Beveridge et al., 2012). A cruise report from the Ocean Tracking Network in the Sea of Gibraltar from 2005 describes the effects of CPDI in ranging experiments conducted in the Mediterranean Sea. Six moorings with VR2-W and VR4 receivers were deployed at depths between 270 and 280 m. Affixed to additional mooring lines placed at various distance from the receiver were Vemco V9, V13, and V16 acoustic tags with output power ranging between 158 and 165 dB. While the depths of tags and receivers are unclear, figures indicate a radial increase in the size of the region impacted by CPDI corresponding to tags with higher power outputs (Beveridge et al., 2012). The positive relationship between the signal strength of tag output and the size of the area affected by CPDI is consistent with expectations from the passive sonar equation.

**Figure 1:**
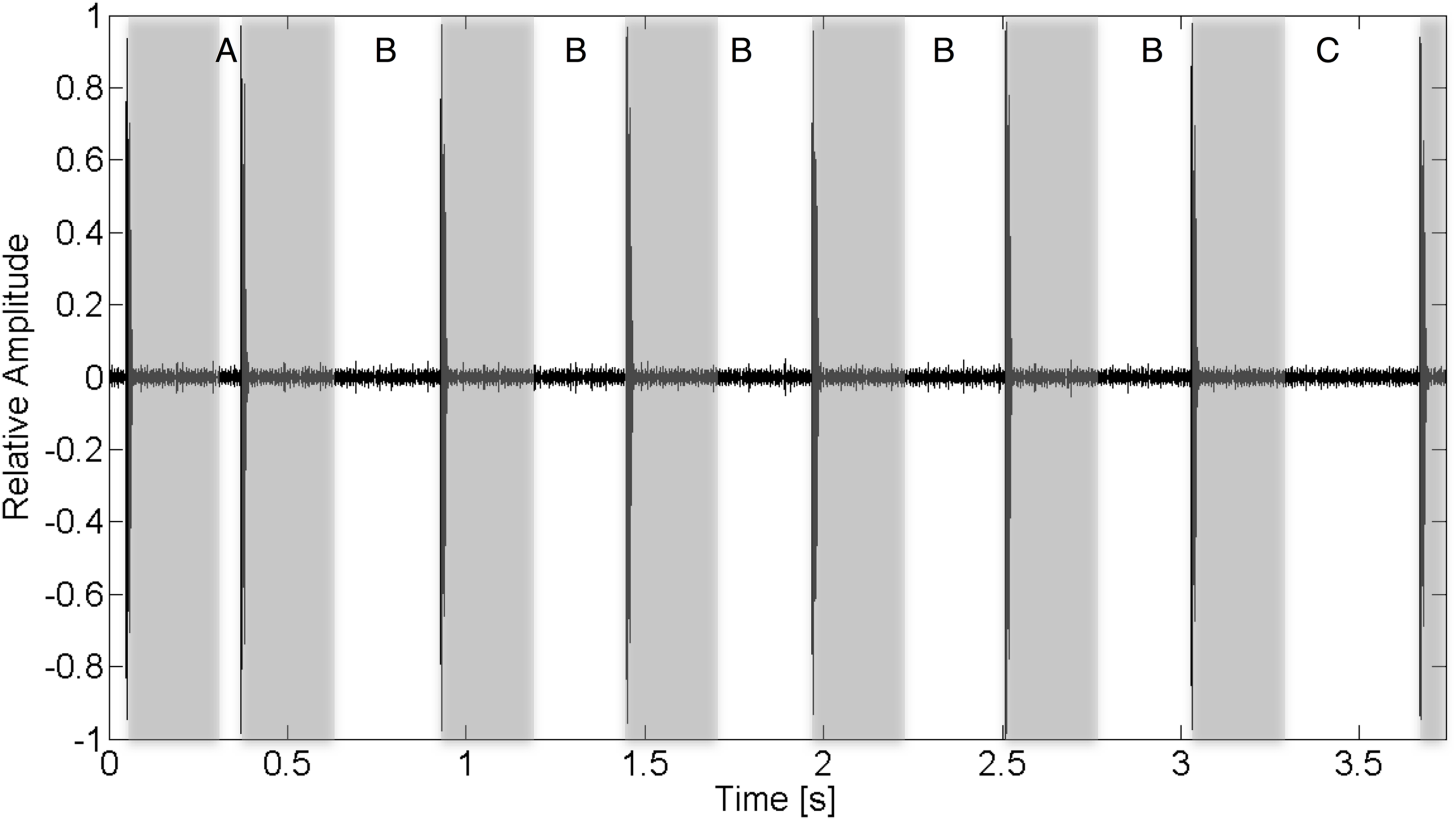
Recorded acoustic waveform of V13 tag transmission indicating the function of various inter-ping interval regions. For this tag, a full transmission train is composed of eight pings. The inter-ping region (A) is the transmission’s synchronization interval. (B) Regions encode the transmitter’s ID. The final interval, (C), is the check sum validation. Gray bars overlaid on the wave form represent a 260 ms blanking interval following the arrival of a ping during which additional acoustic energy arriving at the receiver is ignored. Multipath acoustic energy arriving at the receiver outside of these blanking periods may result in CPDI if the arriving intensity exceeds the detection threshold.

To understand when and how CPDI occurs, it is helpful to understand the way Vemco tags encode and transmit data and how receivers decode and interpret those transmissions. Each transmission consists of a train of 7–10 rapid high-frequency acoustic pings with data encoded in the timing of the intervals between successive pings. The interval between the first two pings, known as the synchronization interval, defines a narrow range of possible coding schemes indicating the tag’s model, a range of potential identification numbers, and other associated data. The last interval acts as a checksum used to confirm that a series of detected pings are from a single train of a valid tag. The remainder of the inter-ping intervals encode the tag’s unique identifier and any sensor data. Each complete transmission lasts roughly 3–5 s (Pincock, 2008). On receipt of each ping, the receiver enters a short “blanking interval” period during which it does not detect additional pings. A blanking interval can have a maximum duration of 260 ms and can be selected by the user during receiver initialization (Fig. 1). When a receiver unit successfully detects the full ping train, including valid synchronization and checksum intervals, it stores the date, time, tag’s unique identifier, and any data from the tag’s environmental sensors (Simpfendorfer, Heupel & Collins, 2008). Acoustic energy in the same operational frequency as the tag arriving at the receiver after the blanking interval and before the subsequent ping may result in failure of the receiver to log the detection or accurately record the tag’s identifier (Simpfendorfer, Heupel & Collins, 2008; Pincock, 2012).

In this manuscript, we will use the term “multipath” in place of “echo” to refer to arrivals of the signal that have been reflected off the sea surface and/or seafloor, for reasons of clarity and consistency with acoustic terminology. CPDI occurs when a ping’s multipath arrives at a receiver during the tag’s transmission sequence, outside of a prescribed blanking interval. If the RL of the multipath is sufficiently high, the receiver may misinterpret the multipath as the arrival of the subsequent ping, resulting in rejection of the transmission (Pincock, 2012; Kessel et al., 2015). The arrival time of each multipath can be calculated from the geometry of the relative position of the tag and receiver in an environment, and the sound speed of that environment. As acoustic energy radiates outward from the tag during each transmission, it can arrive at a receiver via the shortest and most direct path as well as by reflecting off one or more surfaces before arriving at the receiver. The paths of the reflected acoustic energy are termed multipaths. The length of multipaths intersecting the position of a receiver are by definition longer than the direct path, having had to reflect off of some interface during propagation. The relative arrival time of each multipath is therefore a function of the length of the direct path, the multipath propagation distance, and the speed of sound, which itself is dependent on the water’s pressure, salinity, and temperature (Medwin & Clay, 1998).

**Figure 2:**
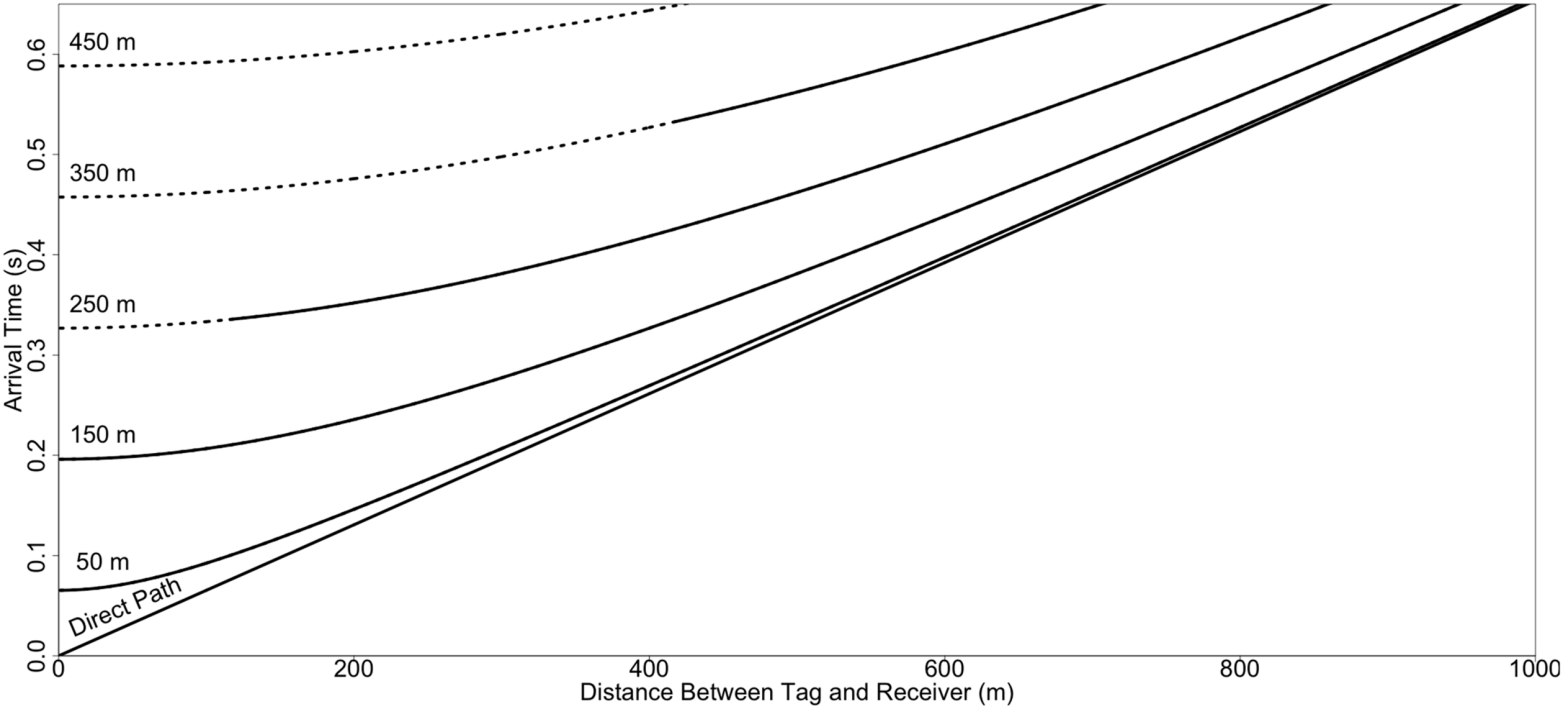
Simulated arrival times for a transmission between a tag and receiver as a function of depth and distance. Arrival time of the direct and first surface reflected multipath. Arrival times were simulated in 100 m increments for depths between 50 and 450 m, with both tag and receiver positioned at the same depth, a fixed sound speed of 1,530 m/s, and an unconstrained (infinite) average maximum detection distance. Dashed lines represent positions of tags and receivers where the arrival of the first surface reflected multipath is predicted to result in CPDI for a receiver with a blanking interval lasting 260 ms. For each depth, as the distance between the receiver and tag increases, the relative arrival time of acoustic energy along the direct path and the first surface reflected multipath converge. CPDI occurs until the point at which the relative arrival time no longer exceeds the blanking interval.

Broadly, reflections result when acoustic energy encounters sharp acoustic impedance contrasts such as those occurring between the water and air and (often to a lesser degree) between water and the seafloor. Acoustic energy may arrive at a receiver having been reflected one or more times off such interfaces. For fixed tag–receiver pair depths, the path length difference (hence relative multipath arrival time difference) between direct and multipath arrivals decreases as the range between tag and receiver increases (Fig. 2). Consequently, increasing tag–receiver separation decreases the number of multipaths arriving after the receiver’s blanking interval, decreasing the likelihood of transmission rejection. Furthermore, the intensity of the reflected signal is attenuated during propagation, with signal strength inversely related to multipath length, resulting in such a point that the intensity of the received signal is no longer exceeds the receiver’s DT. This explains why effects of CPDI are most pronounced at close ranges and only under certain (e.g., reverberant environment) deployment conditions. The goal of the present study is to construct and validate a mechanistic model for CPDI which simulates multipath arrival under various deployment scenarios and can be used to understand and predict when transmission detection may be affected by CPDI. Prior models have been developed to explain the inverse relationship of detection probability and distance (How & de Lestang, 2012; Gjelland & Hedger, 2013) but no other model has considered CPDI. We propose a simple position-based mechanistic CPDI model based on the time delay between direct path transmission and reflected (multipath) arrivals. Our model is based on the hypothesis that a multipath from a tag ping reflected off the sea surface and/or seafloor, arriving after the receiver’s blanking interval with sufficient energy for detection, will cause the receiver to reject the transmission. The purpose of our proposed mechanistic model is to predict when CPDI may result in the rejection of tag transmissions for a given environment and receiver position using parameters commonly derived during equipment ranging experiments. This will allow future studies to use their own range test results to select deployment configurations that mitigate CPDI conditions.

**Figure 3:**
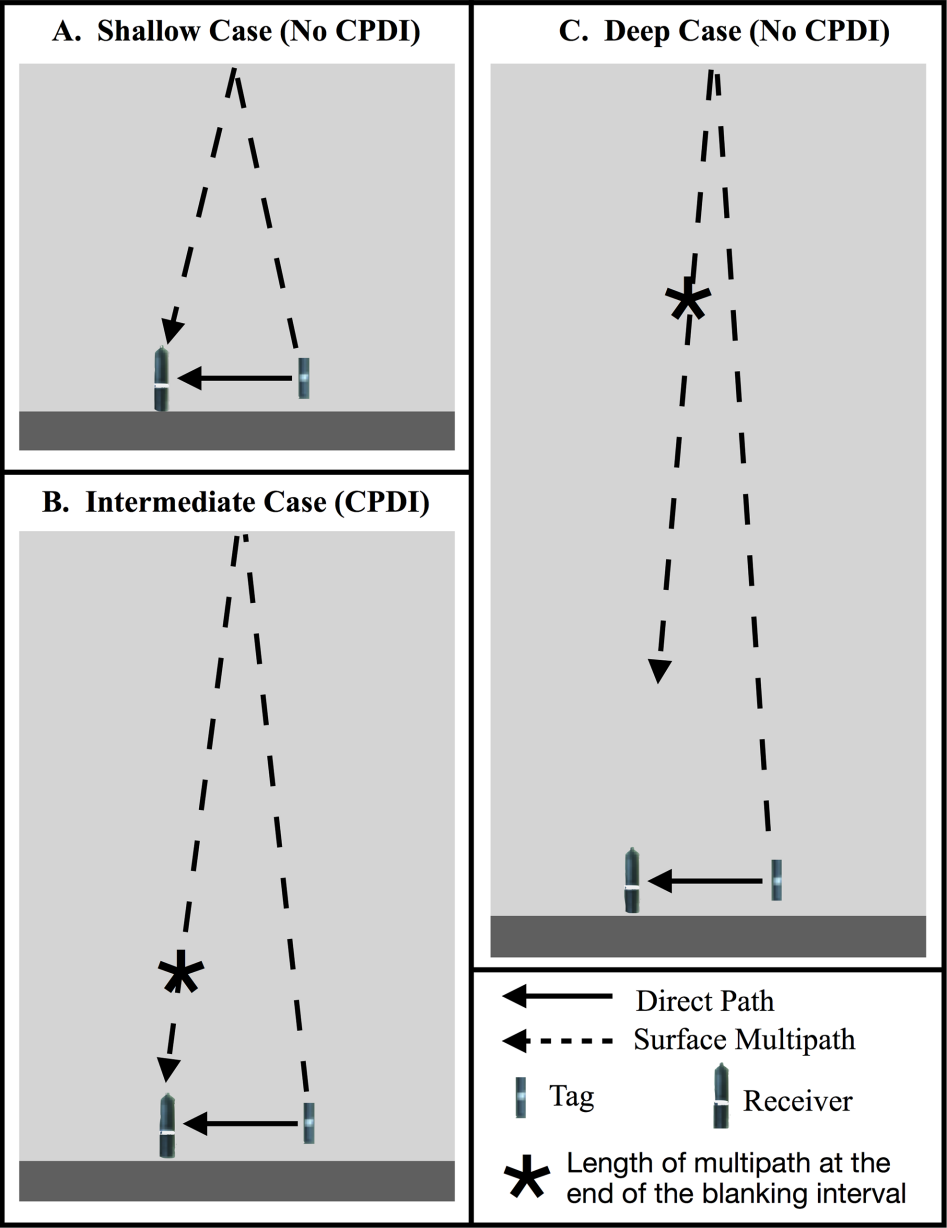
Schematic showing the CPDI outcome of direct and surface reflected multipath arrival as a function of depth. In the simplified scenario considering only the direct and surface reflected multipath, (A) when receiver and tag are sufficiently shallow that the multipath arrives before the conclusion of the blanking interval, the multipath does not result in CPDI. (B) At intermediate depths, the multipath arrives at the receiver following the end of the receiver’s blanking interval, producing CPDI. (C) In environments of sufficiently deep depth, where the path length of the surface reflected multipath is greater than the maximum distance the receiver can detect a tag, the reflected multipath does not arrive with sufficient intensity, and does not result in CPDI.

Our model identifies deployment depth as an important factor contributing to CPDI. Consider the simplest case of the arrival of transmission energy along the direct path and the first multipath reflected off the sea surface in an environment with a uniform sound speed (sound speed is constant across all water depths) where arrival time is directly related to propagation distance of the direct and multipath. When the water surface is smooth, the sea-surface acts as a near perfect reflector with virtually no TL (Urick, 1967). In the case of a sufficiently shallow receiver and tag, the difference in the arrival time of acoustic energy along the direct and surface reflected multipath is less than the receiver’s blanking interval (Fig. 3A). The multipath arrives during the receiver’s blanking interval and does not interfere with the transmission. Holding the horizontal distance between receiver and tag fixed while increasing their depth increases the arrival time difference between the direct and surface-reflected arrival. At sufficient tag/receiver depths, the surface reflection will arrive after the blanking interval (Fig. 3B). When this happens, the receiver may conflate the reflection for the next ping in the transmission resulting in CPDI. Further increasing the depth of the tag and receiver will eventually lead to the point at which the propagation distance for the surface reflection is long enough (i.e., TLs are high enough) that the surface reflection is no longer detectable (Fig. 3C). When this occurs, the reflected ping is not detected by the receiver and CPDI does not occur. This needs to be a consideration as the number of acoustic tracking studies taking place in deeper environments grows.

With this study we conducted a series of sequential experiments building on the results of one another to answer the following questions: How does the shape of the detection function differ between receivers that experience CPDI and those that do not? What causes CPDI? Can we accurately predict where CPDI will occur? How does depth contribute to the CPDI phenomena and what depths are most susceptible?

## Materials and Methods

### Summary

**Figure 4:**
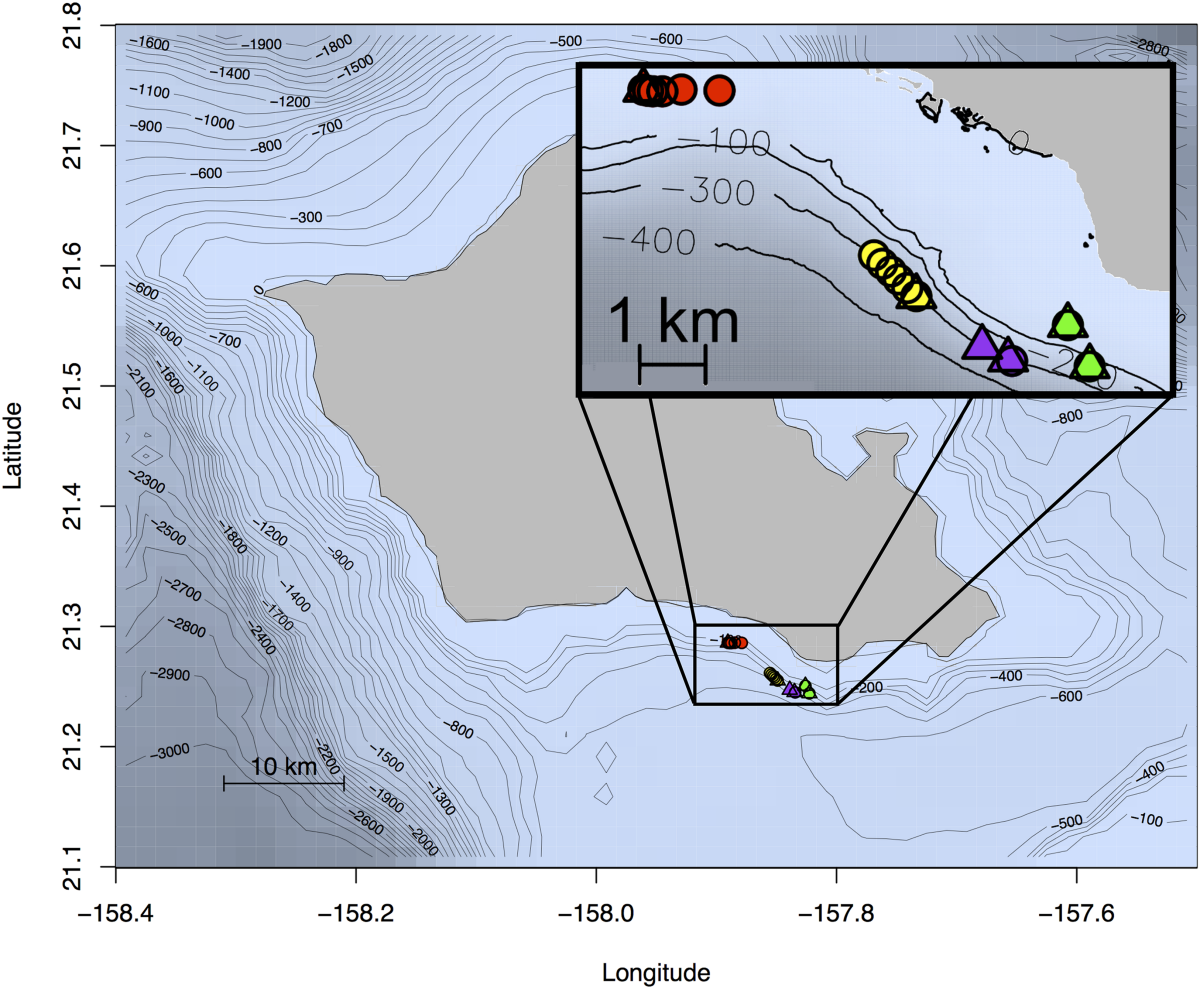
Map of Oahu, Hawai‘i depicting the location of experiments 1–4. The location of each of the four field experiments conducted off the south shore of the island of Oahu, Hawaii. Receiver locations are indicated by triangles and tag locations with circles. Color corresponds to one of the four experiments with yellow showing the location of the deep water ranging experiment (experiment 1), red showing the location of the shallow water ranging experiment (experiment 2), the depth-dependent model validation experiment (experiment 3) in green, and the depth and distance validation experiment (experiment 4) in purple. Bathymetry data from Smith (2016) and Richards et al. (2017).

We performed a series of five experiments which incrementally build on the results of the prior to construct and validate our mechanistic CPDI model. The goal of the first experiment was to determine the range of distances from a receiver at which tags could be detected in a deep water (300 m) environment. The observation of CPDI in the results of this experiment led us to conduct a second range test in a shallow water (25 m) setting to determine if CPDI effects persisted. From observations of the presence/absence of CPDI in experiments 1 and 2, we developed the mechanistic model for predicting CPDI using a simplified straight-line ray-propagation model where direct and multipath arrivals are modeled as a function of sound speed, water depth, and relative receiver and tag positions. We initialized our mechanistic model with similar conditions from the results of experiments 1 and 2 and compared the observed presence and absence of CPDI during these experiments to the mechanistic model’s predictions We then developed two further field experiments comparing CPDI observations with the mechanistic model’s predictions. Finally, we used playback of a recorded acoustic tag transmission in a controlled tank setting to confirm the multipath hypothesis that arrivals occurring after the blanking interval result in missed detections (hence CPDI). The location of each of the four field experiments is shown in Fig. 4. Each experiment is described individually in greater detail in the sections that follow.

### Acoustic telemetry system and generalized performance analysis

Following the work outlined by Kessel et al. (2015), Vemco VR2-W acoustic receivers were used for all experiments. After each experiment, detection logs (with detection time and tag id for all ping train transmissions detections) were downloaded from each receiver using Vemco’s VUE database application and exported as CSV files for further analysis in R (R Core Team, 2014). Except where noted, all experiments used Vemco V13 acoustic tags (69 kHz, 153 dB re 1 μPa @ 1 m) with a variable transmission interval (the time between subsequent ping train transmissions) ranging between 30 and 90 s (60 s nominal transmission interval).

**Figure 5:**
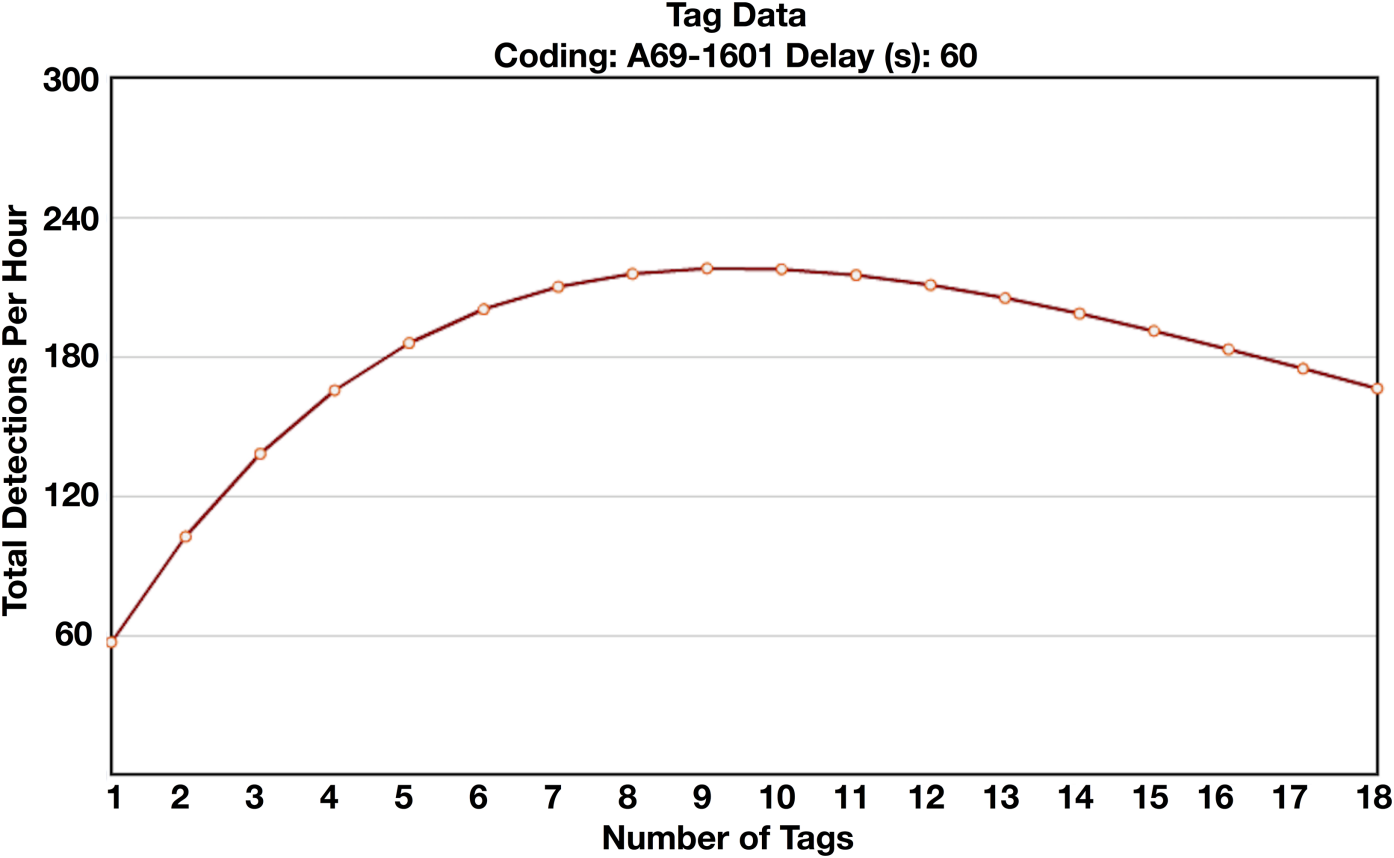
Vemco collision calculator results. Vemco collision calculator results showing the expected number of total detections recorded by a receiver per hour as a function of the number of tags present (Vemco, 2017). As the number of tags detectable by the receiver increases, the probability of overlapping transmissions from multiple tags increases, leading to the rejection of both transmissions. Results shown are for tags with A69-1601 coding scheme and a 60 s nominal delay, the same parameters used in experiments 1–3.

At a glance, the number of detected tag transmissions is significantly lower than would be expected during the first two ranging experiments. This is due to the number of tags used during these experiments and their transmission interval. As the number of tags with variable transmission intervals detectable by a receiver increases, so too does the probability that individual transmissions from two or more tags will overlap. When this occurs, the receiver will reject both transmissions. Therefore, when multiple tags are within the detection range of a receiver, even when transmissions were theoretically detectable on their own, the realized number of detections will be less than the total number of transmissions sent by all tags. This problem is exacerbated when the transmission interval of tags is short, further depressing the number of transmissions detected. For this reason, we present the number of total detections logged by receivers during each hour of the experiment without standardizing values by average number of detections sent per hour as this would be dependent on the exact detection characteristics during each transmission. Vemco’s website provides a collision calculator for estimating the expected number of detectable transmissions when a number of tags with similar transmission parameters are within detection range of a receiver, the results of which we have provided for reference (Fig. 5).

### Experiment 1: quantifying detection range in deep water: 7 June–16 June 2014

A ranging experiment was initially conducted to quantify detection probability at various distances from a receiver for a tracking study investigating the movements of a Hawaiian deep water demersal snapper. The experiment occurred offshore of the Diamond Head crater on the south shore of Oahu. This area was selected as a study site for its accessibility, moderate slope, and similarity to a nearby site involved in other ongoing passive telemetry work. It features a protruding flat shallow shelf between 0 and 100 m extending approximately 1.8 km offshore and terminating with a moderate slope to 700 m over a distance of 5 km into the Kaiwi channel between the islands of Oahu and Molokai (Johnson & Potemra, 2011).

**Figure 6:**
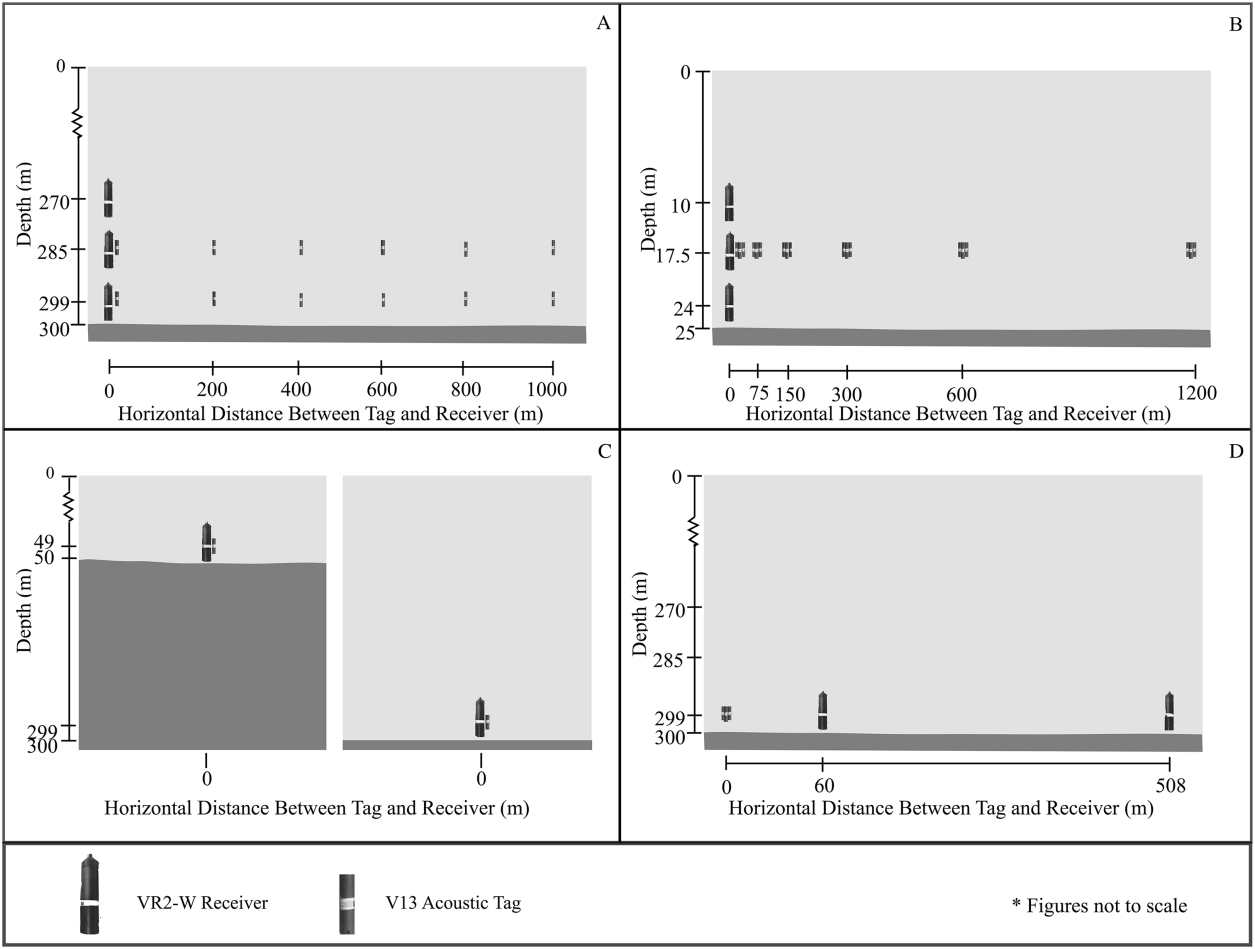
Design of experiments 1–4. (A) Setup of the first component’s deep water ranging experiment was designed to determine AMDR and CPDI extent for a deep water environment. The battery for the receiver positioned 15 m above the seafloor failed, resulting in detection records from the receivers 1 and 30 m above the seafloor only. (B) Setup of the second component’s shallow water ranging experiment, designed to determine AMDR and investigate CPDI in a shallow water setting. (C) The third component’s depth-dependent validation experiment was conceived to validate the predictions of CPDI provided by the mechanistic model with two receiver and tag pairs at different depths. The mechanistic model predicted the effects of CPDI observed by the deeper receiver while no CPDI was predicted for the shallower receiver. (D) The third component’s depth and distance validation experiment was again designed to test the predictive capabilities of the mechanistic model. Two VR2-W receivers were deployed at distances from three acoustic tags. The mechanistic model predicted the receiver closer to the tags but within range of the CPDI affected region would detect fewer transmissions than a receiver farther away and outside the CPDI affected region.

Three receivers were deployed from the R/V Ho’okele in 300 m depth. Receivers were suspended 1, 15 and 30 m from the seafloor on a single mooring using trawling floats, 80 kg of concrete, a polypropylene line, and an acoustic release (LRT; Sonardyne, Yateley, UK). Acoustic tags were moored in a similar manner at 1 and 15 m above the seafloor at ranges spaced by approximately 200 m from 0 to 1,000 m (Fig. 6A). Equipment was recovered 13 days after deployment by activating the acoustic releases. Due to a battery failure in the receiver positioned 15 m off the seafloor, only data from the receivers positioned 1 and 30 m above the seafloor was recovered.

A transmission’s detection probability across the full range of the study was estimated using a generalized additive model (GAM) to explain the number of hourly transmissions detected for each tag and receiver pair as a function of the distance between tags and receivers and the height of the receiver relative to the seafloor, as well as a number of random factors identified by other studies to affect detection distance, using a Poisson distribution to model the error distribution. Random effect variables including mean hourly wind speed and mean hourly wind gust (from NOAA buoy #161234), hourly tide height and hourly tide direction data (from NOAA tide station #1612340), and diurnal period, divided into day (6am to 6 pm) or night (6 pm to 6 am). GAMs were fit using the Mixed GAM Computational Vehicle (mgcv) package in R (Wood, 2011). From GAM results, the number of transmissions detected was predicted for all distances up to the maximum tag range in 1 m increments and then used to determine AMDR and the extent of the area from the receiver affected by CPDI. The distance variable was fit with a penalized regression spline smoother, selected to reduce the potential of overfitting the data when estimating the number of detections between sampled ranges. The largest appropriate basis dimension, 6, was selected for the smoother argument to minimize the underfitting bias of the region closest to the receiver, where CPDI has the potential to occur, by detections from tags at ranges unaffected by CPDI. All random effects were fit with a ridge penalized smoother and the value of the basis term for each was assessed for statistical appropriateness.

From the resulting global GAM, candidate GAMs consisting of all possible permutations of independent explanatory variables were compared to determine the best fit models using Akaike’s information criterion (AIC). Candidate models within two AIC units of the best fit GAM were used to estimate AMDR and CPDI extent. The number of expected hourly detections across the range of potential tag locations for each combination of explanatory factors were predicted using each GAM using median values for wind speed, wind gust, water level, and incoming/outgoing tides during both day and night periods. Predicted hourly detections were then used to determine AMDR and presence/extent of CPDI. AMDR was defined as the distance at which the number of detections predictedfell below a threshold of 5% of transmissions sent. In practice, this occurred when there were fewer than three predicted detections per hour. We then constructed a range including standard error around this value by also predicting the standard error values at each predicted distance and then adding and subtracting the error from our model fit. We then calculated a range inclusive of the standard error as the distance where each of our predictions incorporating the error term fell below our 5% threshold as a measure of the model’s fit. CPDI was said to affect the range from the receiver to the distance at which the predicted number of detections and their standard error first overlapped the maximum predicted value and its standard error. At this point we could be 95% confident the predicted values no longer statistically differed.

### Experiment 2: quantifying detection range in shallow water: 22 November–2 December 2014

A second experiment was designed to determine the relationship between detection probability and horizontal distance in a shallow water setting, and to explore whether CPDI was present in this setting. A field site was selected off Sand Island, immediately west of the Honolulu Harbor channel. Characterized by a loose sand substrate and sparse coral rubble, this location was selected for accessibility to a relatively linear swath of 25 m isobath, water properties presumed similar to the deep water ranging experiment site due to their geographic proximity, and a standing agreement between the University of Hawaii and Hawaii’s Department of Aquatic Resources for use of the area for research purposes.

Nine Vemco VR2-W units were deployed on a single mooring from the R/V Hoʻoponopono. The mooring design used was similar to the one employed in the deep water ranging experiment except that the polypropylene line was reinforced with a 1/8″ braided steel cable and acoustic releases were not used. The nine receivers were suspended in groups of three at 1, 7.5, and 15 m above the seafloor. Eighteen acoustic tags were affixed 7.5 m from the seafloor, in groups of three, spaced at approximate horizontal distances of 0, 75, 150, 300, 600, and 1,200 m from the receivers, as measured by GPS during each mooring deployment (Fig. 6B).

Following deployment, divers descended on the receiver mooring to assess equipment condition and measure the bottom depth which was found to be 25 m using a dive computer (Zoop; Suunto, Vantaa, Finland). Bottom depth was measured using the same dive computer during recovery of the tag moorings which ranged between 23.8 and 25.3 m. The same process for determining AMDR and CPDI extent was performed for data from this shallow water ranging experiment as was done during the deep water ranging experiment.

### Development of a mechanistic model for predicting CPDI

**Figure 7:**
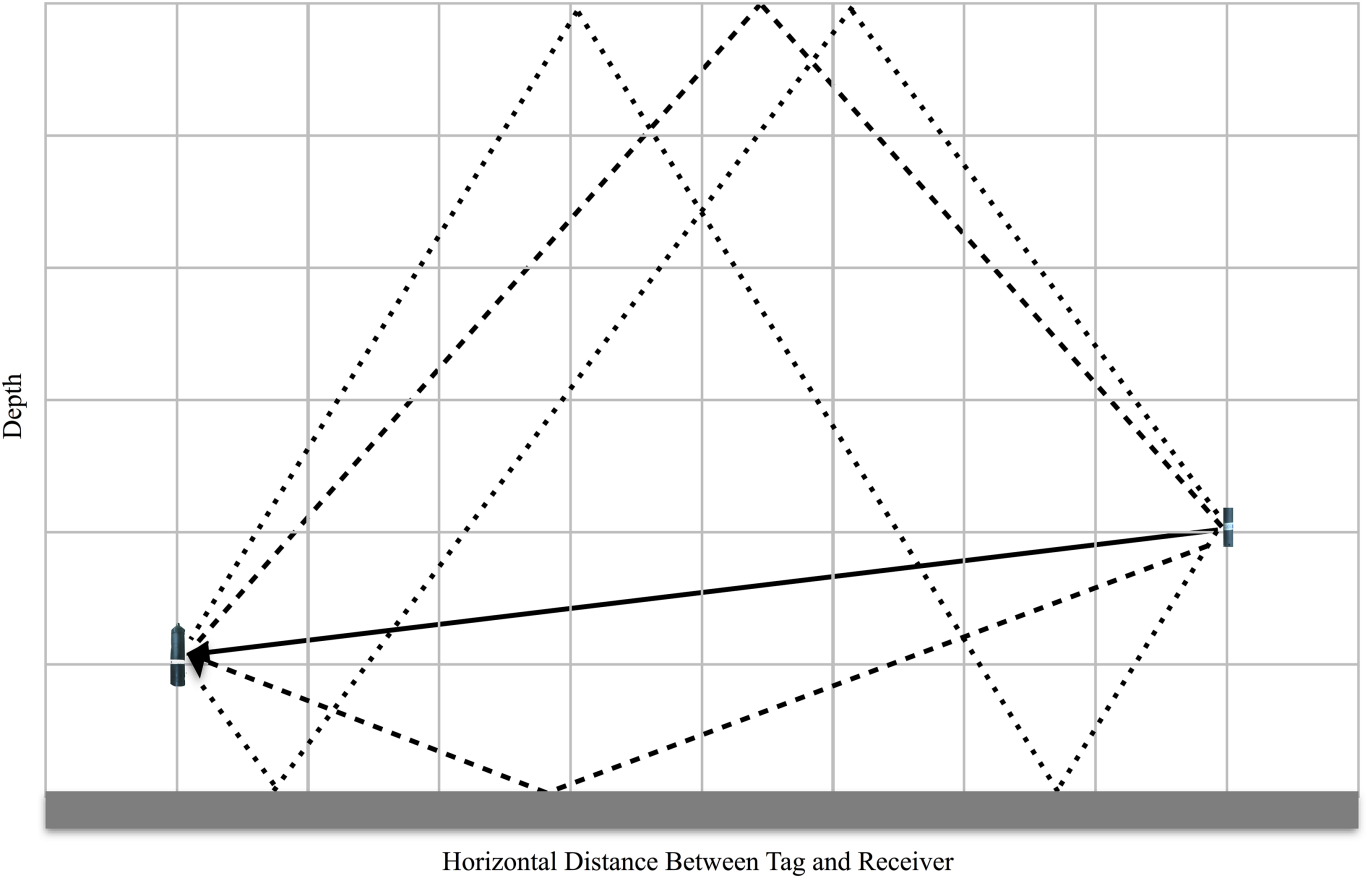
Sketch of the mechanistic CPDI model applied to a hypothetical environment. The direct transmission path from source to receiver is represented by solid arrow and the first four multipath arrivals reflecting off the surface and seafloor are illustrated with dotted arrows. With the assumption of a uniform sound speed, the arrival time of the direct arrival and each multipath is a function of their respective path length. When the difference in path length between any multipath and the direct path is greater than the product of the speed of sound and the receiver’s blanking interval, CPDI is predicted to occur.

The proposed mechanistic CPDI model uses a depth and range-independent sound speed (i.e., straight-line acoustic propagation), relative positions of the receiver and tag, water depth at the receiver, the duration of the receiver’s blanking interval, and AMDR determined from ranging experiments to calculate the path length of direct and multipath arrivals for a grid of potential tag position (Fig. 7). All direct and multipath arrivals with a path length less than or equal to the AMDR are considered by the model. Our model assumes that the only factor affecting detection of acoustic energy by the receiver is the length of the propagation path. Our model does not account for scattering and reflective losses at the surface and seafloor (i.e., we assume TLs are equal for equal path lengths regardless of propagation path). Since some energy loss is always incurred on reflection, this approach considers the multipath arrivals that in practice may not be detectible by the receiver. This results in the potential for falsely predicting CPDI observations where they may not be present in an experimental setting, resulting in a more conservative model with predictions of a “worst-case scenario” situation. However, when surface conditions are calm, TLs at the sea surface are nominal (Urick, 1967). Our model also cannot account for minor variation in tag output as a result of tolerances in Vemco’s manufacturing process. Implementations of our model, in both R and Matlab, are provided as Supplemental Information.

The first step of the proposed mechanistic model is to grid the study area by range and depth, with each grid point representing a potential tag position and the receiver fixed at 0 m range. A resolution parameter allows the user to select an appropriate grid spacing. A ray tracer calculates both direct and multipaths lengths at each grid point using an ideal model of multipath propagation (Lurton, 2010). This is repeated for each multipath until a set of all multipath lengths less than the AMDR is compiled. Our model then predicts the occurrence of CPDI by evaluating the propagation path lengths of the direct and multipath arrivals by two criteria. The direct path length is subtracted from the length of each multipath and multiplied by a sound speed constant to determine a relative arrival time for each multipath. The set of relative arrival times for each grid point is then assessed using our two criteria: Do any multipath arrivals have a path length less than AMDR? If so, do these path lengths have relative arrival times greater than the receiver’s blanking interval? The reasoning behind the criteria is as follows: The direct path arrival of the first ping in the tag’s ping train, arriving before any multipath arrivals, should trigger the receiver to begin the blanking interval. Once the blanking interval ends, any detectable multipath arriving (e.g., the surface reflected bounce of the first ping) may cause the receiver to reject the ping train since the receiver is expecting the direct path arrival of the second ping in the train. Rejection is not predicted for multipaths with lengths longer than the AMDR as we assume TLs incurred during propagation will be equal to or in excess of the direct path, and will therefore be undetectable to the receiver.

Therefore, each multipath arriving at a receiver may fall into one of three categories. (1) If the relative arrival time is less than or equal to the blanking interval and the total path length is less than or equal to the AMDR, the multipath is not predicted to interfere with detection of the direct signal. (2) If the relative arrival time is greater than the blanking interval and the total path length is less than or equal to the AMDR, the multipath is predicted to interfere with the direct signal resulting in failure of the receiver to detect the transmission. (3) If the path length is in excess of the AMDR, no interference is predicted, as the multipath has experienced TLs during propagation such that it is below the threshold for detection. At each grid point, each multipath is categorized. Grid points with at least one multipath falling into the second category are predicted to experience CPDI based on our criteria; grid points where all transmission multipath are of the first and third type are predicted not to experience CPDI.

Following the development of the mechanistic CPDI model, we input parameters from both deep water and shallow water ranging field experiments to compare the observed ranges affected by CPDI to predictions from the mechanistic CPDI model. We used a 260 ms blanking interval (by default the longest blanking interval available when initializing a VR2-W), a sound speed of 1,530 m/s (typical of the environment in which testing was performed (Tsuchiya et al., 2015)), and a grid resolution of 1 m. For the deep water ranging experiment (experiment 1), transmission detection was predicted by simulating receivers at 270 and 299 m depth in a water column depth of 300 m across horizontal distances up to 1,000 m from the receiver with the mechanistic CPDI model. For the shallow water range experiment (experiment 2), receivers were simulated at 24, 17.5, and 10 m depth in a 25 m environment over the 1,200 m range tags were deployed. The AMDR variable was defined as the distance at which the number of transmissions detected by receivers, estimated from the median of all considered candidate GAM estimations, fell below 5%. With a nominal transmission interval of 60 s, this threshold was three detections per hour for the tags used in both experiments.

### Experiment 3: depth-dependent model validation: 17 March–25 March 2015

The first of two validation experiments was designed to test predictions of CPDI related to deployment depth. In this experiment, the mechanistic CPDI model was used to identify two depth conditions: One in which multipaths were predicted to arrive outside the receiver’s blanking interval, producing CPDI, and a second, where no detectable multipaths arrived outside the receiver’s blanking interval, and thus no CPDI effects were present. The mechanistic model’s AMDR parameter was set to 843 m, the closest whole number to the median value determined during the deep water ranging experiment (experiment 1), due to similarities in depth and deployment location. The model’s blanking interval was initialized at 260 ms and sound speed was 1,530 m/s. The mechanistic model predicted CPDI for receiver and tags on the same mooring line (a horizontal distance of 0 m), when both receiver and tag were positioned 1 m above the seafloor in 215 m bottom depth. No CPDI was predicted for a similar tag and receiver pair in 50 m water depth. Latitude and longitude coordinates were selected for locations matching these depths in proximity to the location where the deep water ranging experiment was conducted using bathymetry charts (Johnson & Potemra, 2011). One mooring was deployed at each site from the RV Hoʻokele. Each of the moorings consisted of a tag and receiver positioned 1m from the seafloor. The vessel’s depth sounder indicated that the unit intended for deployment at 50 m was deployed at its target depth, while the receiver intended for 215 m was deployed just off target in 212 m water depth (Fig. 6C). The experiment ended prematurely when the 50 m unit broke free of its mooring and was recovered by State of Hawaii Division of Aquatic Resources enforcement officers nine days after deployment. Logistics and strong trade wind conditions prevented recovery of the remaining unit for a further eight weeks.

The number of tag transmissions detected hourly by each receiver was assessed for normality using Shapiro–Wilks’ test and were compared between receivers using a Wilcoxon sign-rank test due to the nonparametric distribution of data collected. To account for the independence in the number of transmissions sent by each tag, daily meta-logs for each receiver were downloaded from the VUE database. These provided the number of valid detections, valid synchronization intervals, total detected pings, and the number of detections rejected due to invalid checksums logged by each receiver. Daily performance metrics, including code detection efficiency (CDE) and the rejection coefficient (RC) were determined for each receiver from meta-logs using methods previously established (Simpfendorfer, Heupel & Collins, 2008). CDE is defined as the fraction of detected transmissions to the number of detected first inter-ping intervals (synchronization intervals). CDE ranges between 0 and 1, and is a measure of the receiver’s ability to successfully record a detected transmission. RC is the fraction of transmissions rejected for failure to validate the checksum relative detected synchronization intervals (Simpfendorfer, Heupel & Collins, 2008).

These metrics allowed receiver logs to be normalized for comparison independently of the total number of tag transmissions sent. This is important when comparing detection logs in which variations in transmission interval may have resulted in each receiver being exposed to a different number of transmission ping trains. However, both CDE and RC use the number of detected valid syncs as a proxy for the number of transmissions sent. For a receiver to recognize a synchronization interval, the time between the arrival of two pings must be of a strictly defined length. We suspect multipath arrivals of the first ping of the synchronization interval may occur before the subsequent ping, resulting in failure of the receiver to categorize these pings as defining a valid synchronization interval. If this occurred, the number of synchronization intervals would be an underestimate of the number of transmissions for a receiver experiencing the effects of CPDI. To decouple our CDE and RC receiver metrics from the number of synchronization intervals, we created adjusted CDE and RC metrics replacing the number of detected syncs with number of pings detected reduced by a factor corresponding to the number of pings composing a complete transmission train. For our tags, a complete transmission train consisted of eight pings.

### Experiment 4: depth and distance model validation: 25 May–30 May 2015

The second of the validation experiments was designed to test the mechanistic CPDI model with respect to depth and distance. Simulations using the mechanistic CPDI model indicated that in 300 m water depth, multipath arrivals producing CPDI conditions would persist to distances of 255 m when receivers and tags were positioned 1 m above the seafloor using a sound speed of 1,530 m/s and an 843 m estimate for AMDR. Therefore, it was predicted a receiver positioned 500 m from a group of tags would be more likely to detect a greater number of transmissions than a receiver positioned 50 m from the same tags, within the range CPDI was predicted. Three acoustic tags with 15 min fixed transmission intervals were activated 5 min offset from one another to prevent transmission overlap and moored off Diamond Head in 300 m of water. Two separate VR2-W moorings were deployed at target distances of 50 and 500 m from the transmitter tags along the 300 m isobath. GPS marks taken during deployment indicated the receiver targeted for 50 m was deployed 10 m off mark, 60 m from the tags, and that the receiver targeted for 500 m was deployed 8 m off mark, 508 m from the tags (Fig. 6D). The normality of hourly recovery rate data was again assessed for each condition using Shapiro–Wilks’ test and then between conditions using a Wilcoxon sign-rank test.

### Experiment 5: multipath confirmation: 13 July 2016

A controlled tank experiment was designed to test the underlying hypothesis behind our CPDI model, that the primary driver of CPDI is spurious ping multipaths arriving after the blanking interval. A laptop running Matlab’s Data Acquisition Toolbox (MathWorks 2015) was used to playback a waveform signal recorded from a V13 acoustic tag using a digital-to-analogue converter, amplifier, and two ITC 1042 transducers (one transmitting and one recording the sound) with a relatively flat sensitivity of −200 dB re 1 V/μPa between 1 and 100 kHz and a sampling frequency of 192 kHz (we refer to the transmitting and recording transducers as the “transmitter” and the “hydrophone,” respectively). The transmitter was suspended in the tank about 1 m away from a VR2-W receiver unit and the hydrophone. The output level of the transmitter was calibrated to match the output of a tag by incrementally increasing amplifier output until the peak-to-peak voltage measured by the hydrophone matched the output level produced by the acoustic tag placed in the tank at the same position as the transmitter.

Recordings of the acoustic tag were processed to create a simulated tag transmission. TL for each simulated multipath was calculated using a straight-line acoustic propagation model to calculate the path length (*I*_Arr_) for each of the first 20 acoustic arrivals (direct arrival and interface-reflected multipath arrivals). Then, the RL factor for each arrival path was calculated using the formula:

$${\rm{RL}} = {10^{ - 1*{\rm{lo}}{{\rm{g}}_{10}}({I_{{\rm{Arr}}}})}}$$

This yielded 20 sets of scalars by which the simulated transmission waveform, was multiplied to get the simulated RL of each multipath determined from its simulated arrival path. These scalars were turned into the impulse response by placing them at the appropriated time delay relative to the direct path arrival time, based on the time of arrival information from the mechanistic model for predicting CPDI. A waveform containing the direct transmission signal, and when appropriate, simulated multipath arrivals, was then constructed by convolving the simulated source waveform with this impulse response. Further reductions in signal intensity for multipath arrivals to mimic TLs incurred during reflection and scattering at surface and seafloor interfaces were not considered. Reflections from the walls of the tank were not expected to produce CPDI as preliminary testing indicated the tank had an impulse response length shorter than the receiver’s 260 ms blanking interval. In other words, the NL in the tank returned to ambient levels within the 260 ms window of the blanking interval.

All permutations of tag and receiver placement from field experiments were simulated with and without multipath arrivals. This led to two conditions: a control condition in which only the direct arrival was emitted into the tank (and thus CPDI not predicted), and an experimental condition which included both the direct path and simulated multipaths. Scenarios in the experimental condition were further categorized into those in which CPDI was predicted and those in which PCDI was not predicted, according to the CPDI model criterion. All simulated transmissions were repeated five times.

Each simulated transmission was assigned an event identification based on the experiment simulated and the placement of the receiver in the water column. One of three predictive classifications were assigned to each transmission: (1) no multipath (control), (2) with multipath, no CPDI predicted, and (3) with multipath, CPDI predicted, leading to four possible outcomes (1) detection predicted, detection occurred, (2) detection predicted, no detection occurred, (3) no detection predicted, no detection occurred, and (4) no detection predicted, detection occurred. A transmission was coded 1 if it was detected by the receiver and 0 if it was not detected. A logistic regression was fit using a generalized linear model (GLM) with transmission detection/nondetection as the binary response variable. Predictor variables included the predictive classification (control, with multipath, no CPDI Predicted, with multipath CPDI predicted), and the event ID representing the analogous experiment and condition simulated. Terms representing the interaction between predicted/observed and each event ID, which would identify any simulated experimental analogues where observations systematically varied from predictions were also considered. Model selection was used to identify the best GLM. A pseudo *R*^2^ was calculated for the GLM (McFadden, 1974) and hierarchical partitioning was performed to determine the percentage each term contributed to the GLM’s overallexplanation of the observed variance.

## Results

### Summary

**Figure 8:**
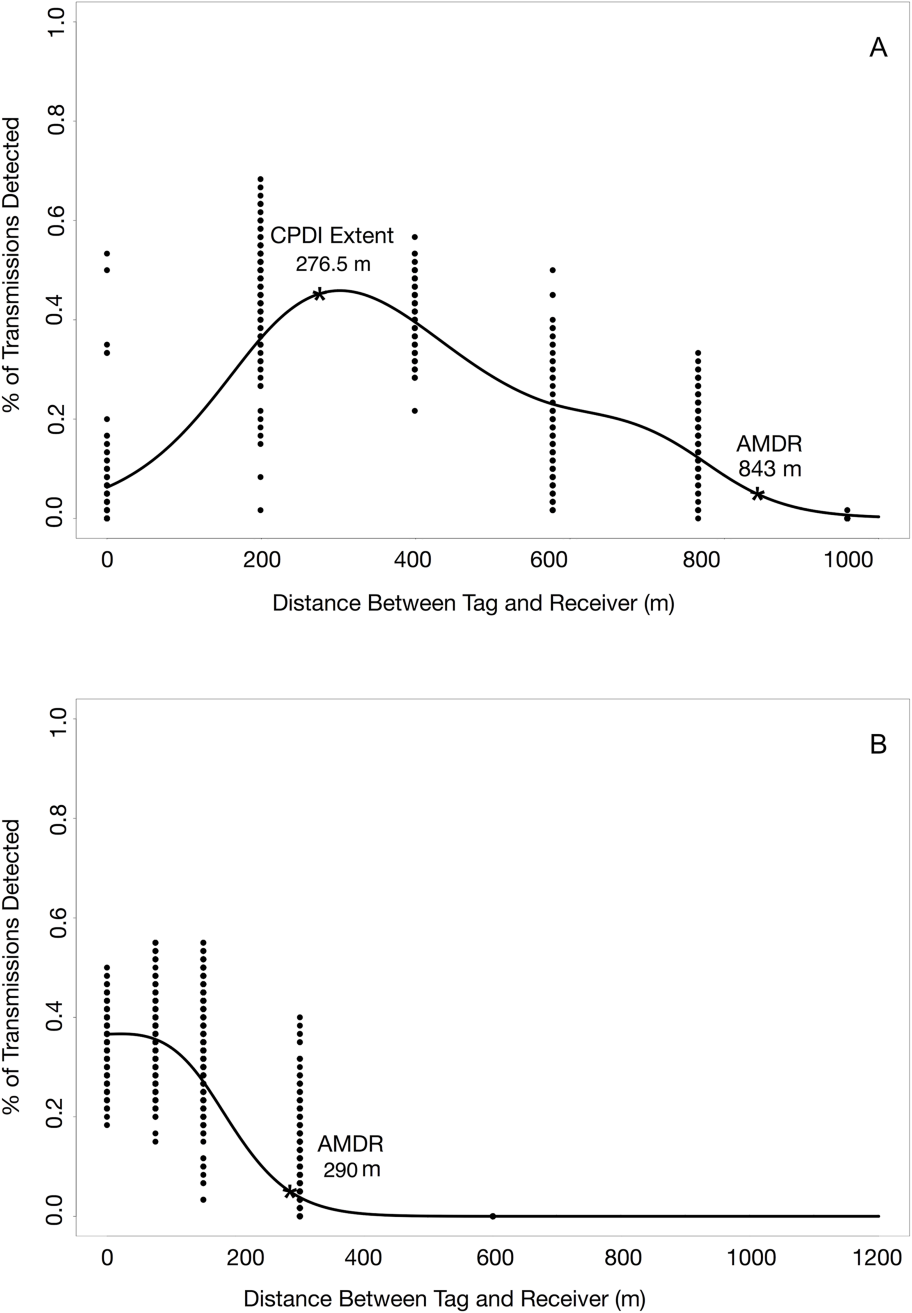
Detection probability profiles from deep and shallow water ranging experiments. (A) Effects of CPDI are clearly present in the results of the deep water ranging experiment, as indicated by low detection probabilities at ranges close to the receiver increasing to a maximum detection probability at an intermediate range. (B) Effects of CPDI are not present in detection probabilities of the shallow water ranging experiment, with the maximum detection probability occurring at distances nearest the receiver.

The shape of the detection functions for the deep water ranging experiment (experiment 1) differed from that of the shallow water ranging experiment (experiment 2) (Fig. 8). The presence of CPDI in the deep water experiment created an area of low detection probability surrounding the receiver, with the highest number of observed detections coming from tags at an intermediate distance from the receivers. In contrast, the highest observed number of detections during the shallow water ranging experiment, where no CPDI was observed, came from the tags positioned closest to the receivers. Our mechanistic model for predicting CPDI was largely congruent with field observations from ranging and validation tests, accurately predicting when the effects of CPDI were observed. For both validation experiments, detection of transmissions from tag to receiver pairs where no CPDI was predicted surpassed those where CPDI was predicted by our mechanistic model. In controlled tank experiments, we were able to accurately predict the detection/nondetection of 460 simulated transmissions with 95.7% accuracy using our multipath arrival prediction criterion.

### Experiment 1: quantifying detection range in deep water: 7 June–16 June 2014

During the deep water ranging experiment, on average, the range at which tag transmissions were detected ranged between 840 and 846 m (range including standard error: 839–847 m) with some variation arising from different factor levels of random predictor variables (Fig. 8A). The range affected by CPDI extended 276.5 m (range including standard error: 276–277 m) from the receiver for all permutations of predictor variables. The influence of each combination of predictor variables on GAM estimates of AMDR and CPDI range are presented in Table 1.

**Table 1:** Deep water ranging experiment results.

Receiver height (m)	Tag height (m)	Tidal phase	Diurnal period	GAM estimated AMDR (m)	GAM estimated CPDI (m)	Model predicted CPDI (m)
1	1	In	Day	843 (839–847)	276.5 (276–277)	259
1	1	Out	Day	843 (839–847)	276.5 (276–277)	259
1	1	In	Night	843 (839–847)	276.5 (276–277)	259
1	1	Out	Night	843 (839–847)	276.5 (276–277)	259
1	15	In	Day	844 (839–847)	276.5 (276–277)	279
1	15	Out	Day	843.5 (839–847)	276.5 (276–277)	279
1	15	In	Night	844 (839–847)	276.5 (276–277)	279
1	15	Out	Night	843.5 (839–847)	276.5 (276–277)	279
30	1	In	Day	844 (842–847)	276.5 (276–277)	301
30	1	Out	Day	845 (842–847)	276.5 (276–277)	301
30	1	In	Night	844 (839–847)	276.5 (276–277)	301
30	1	Out	Night	844 (839–847)	276.5 (276–277)	301
30	15	In	Day	843.5 (842–845)	276.5 (276–277)	326
30	15	Out	Day	843.5 (842–845)	276.5 (276–277)	326
30	15	In	Night	843 (839–847)	276.5 (276–277)	326
30	15	Out	Night	843.5 (839–847)	276.5 (276–277)	326

**Notes:**

Median predictions of AMDR and CPDI from all candidate GAMs and, in parenthesis, the minimum and maximum value predicted by any one candidate GAM inclusive of standard error. Also presented are estimates for CPDI range from the proposed mechanistic model, fit with the median AMDR value for each combination of factors.

There were eight GAMs with AIC values equal to or within two AIC values of the lowest, and thus best fit, model. Each of these explained 64.6% of variation in the number of transmissions per hour detected by the receivers (Adjusted *R*^2^ = 0.647). The predictor variables included in the GAM with the lowest AIC were distance, receiver height, tag height, mean hourly wind speed, mean hourly wind gust, and diurnal period.

### Experiment 2: quantifying detection range in shallow water: 22 November–2 December 2014

During the shallow water ranging experiment, on average, tag transmissions weredetected up to a distance ranging between 278 and 290 m (range including standard error: 277–290 m) from the receiver (Fig. 8B). CPDI was not observed during this experiment; that is, the GAM estimated CPDI was 0. The influence of each combination of predictor variables on GAM estimates of AMDR and CPDI range are presented in Table 2.

**Table 2:** Shallow water ranging experiment results.

Receiver height (m)	Tidal phase	Diurnal period	GAM estimated AMDR (m)	GAM estimated CPDI (m)	Model predicted CPDI (m)
1	In	Day	290 (289–290)	0 (0–0)	0
1	Out	Day	290 (289–290)	0 (0–0)	0
1	In	Night	285 (284–285)	0 (0–0)	0
1	Out	Night	285 (284–285)	0 (0–0)	0
7.5	In	Day	283 (282–283)	0 (0–0)	0
7.5	Out	Day	283 (282–283)	0 (0–0)	0
7.5	In	Night	278 (277–278)	0 (0–0)	0
7.5	Out	Night	278 (277–278)	0 (0–0)	0
15	In	Day	284 (283–284)	0 (0–0)	0
15	Out	Day	284 (283–284)	0 (0–0)	0
15	In	Night	278 (278–279)	0 (0–0)	0
15	Out	Night	278 (278–279)	0 (0–0)	0

**Notes:**

Median predictions of AMDR and CPDI from all candidate GAMs and, in parenthesis, the minimum and maximum value predicted by any one candidate GAM inclusive of standard error. Also presented are estimates for CPDI range from the proposed mechanistic model, fit with the median AMDR value for each combination of factors.

There were four GAMs with AIC scores equal to or within two values of the lowest, and thus best fit, AIC value. Each of these four candidate GAMs explained approximately 72.7% of the variation in the number of detected transmissions per hour (Adjusted *R*^2^ = 0.684). Predictor variables for the GAM with the lowest AIC score included distance, receiver height, diurnal period, mean hourly wind gust, mean hourly wind speed, and mean hourly water level.

### A mechanistic model for predicting CPDI

We input environment parameters from the deep and shallow water ranging experiments (experiments 1 and 2) and their median AMDR estimates into our mechanistic model for CPDI. CPDI estimates from range test results were compared to the mechanistic model’s predictions (Tables 1 and 2). For the deep water ranging experiment, the mechanistic model predicted CPDI extending from the receiver to distances between 259 and 326 m while GAM predictions estimated CPDI extent to 276.5 m from the receiver (Table 1). Predictions of the CPDI ranges using the mechanistic predictive CPDI model were within 52 m of the median estimations from the GAM models for the deep water ranging experiment (experiment 1), differing by an average of 14.75 ± 9.44 m. For the shallow water ranging experiment, CPDI was neither predicted nor observed by either method (Table 2).

As the mechanistic CPDI model does not consider TLs from reflection and absorption, it was not unexpected that the CPDI model predicted a slightly larger CPDI range than that estimated by the GAMs results. Only the combination of receiver and tag both positioned 1 m above the seafloor produced GAM estimated CPDI ranges larger than those predicted by the CPDI model.

### Experiment 3: depth-dependent model validation experiment: 17 March–25 March 2015

During this experiment, observed detections of tag transmissions by each receiver were consistent with predictions made by the mechanistic model. Shapiro–Wilks’ tests indicated that distributions for the number of hourly detections by each receiver were non-normal (*p* < 0.05 for the 50 m case and *p* < 0.001 for the 212 m case). The number of detections recorded by the two receivers differed significantly as determined by using a Wilcoxon sign-rank test (*p* < 0.001). The 50 m tag/receiver pair experienced mean detection rates over 5.5 times greater than that of the 212 m tag/receiver pair (56.6 detections per hour vs. 10.0 detections per hour, respectively). There were no periods in which the deeper receiver, where CPDI producing multipaths were predicted, detected more transmissions than the shallow receiver where CPDI producing multipaths were not predicted.

**Figure 9:**
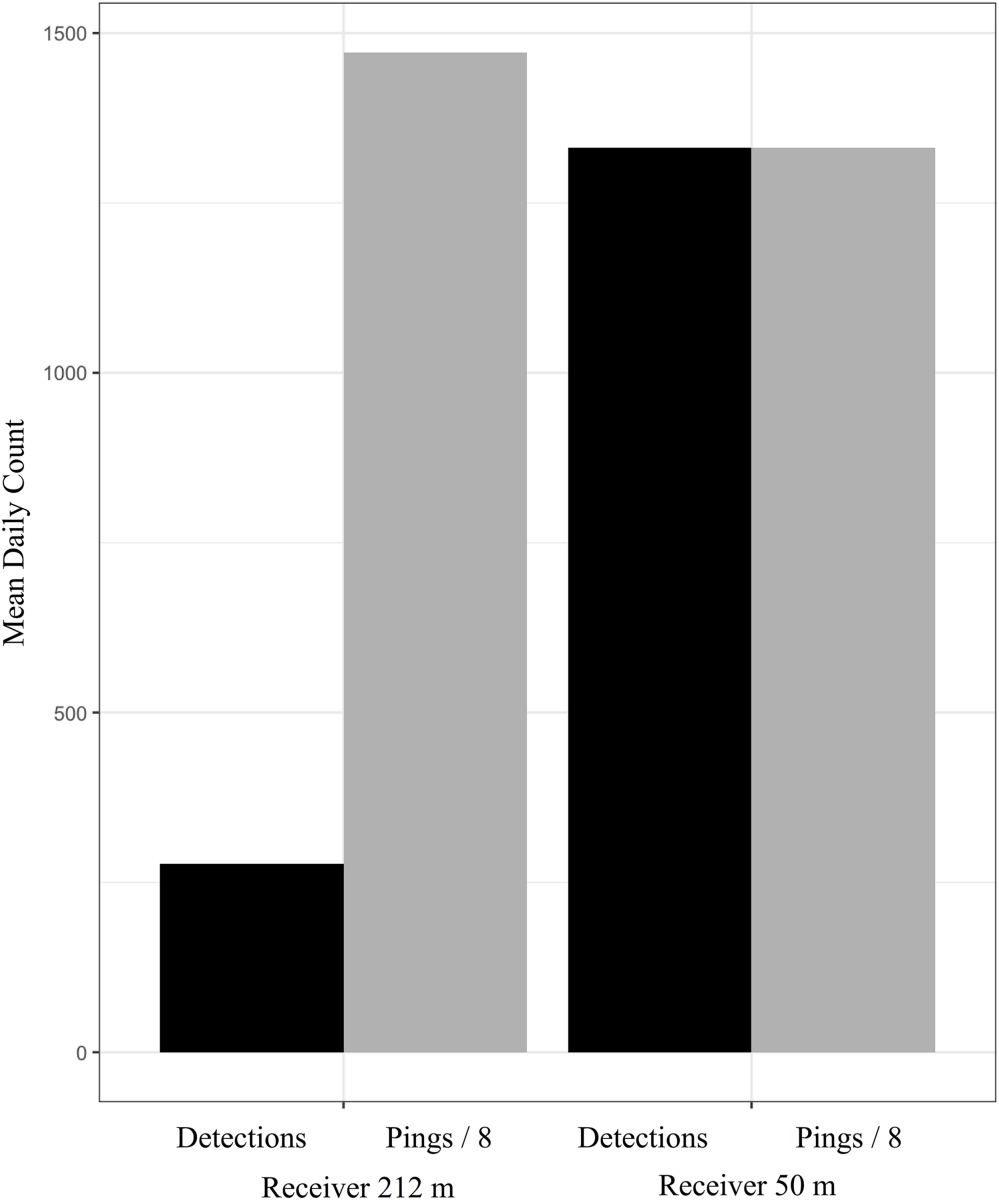
Comparing the mean daily components of the adjusted CDE between receivers in the depth-dependent model validation experiment (experiment 3). The number of pings detected has been standardized by a factor of 8, which is the number of pings comprising a full transmission and a proxy of the total number of transmissions sent. The receiver affected by CPDI (212 m depth) detected a greater number of transmission pings but detected substantially fewer transmissions than the receiver not affected by CPDI (50 m depth).

Assessment of performance data for each receiver from meta-logs was done using conventional CDE and RC metrics with the number of detected syncs serving as a proxy for total transmissions as well as adjusted metrics substituting the syncs for the number of pings detected divided by the number of pings composing a full transmission. For both metrics, nonparametric methods were required due to nonequivalent variances between receivers and a non-normal distribution of both CDE and adjusted CDE from the receiver in 50 m depth. The 50 m depth receiver had median CDE and adjusted CDEs of 1.00 (meaning virtually no detections were missed) while the 212 m receiver had a significantly lower median CDE of 0.0865 (*p* < 0.01; paired Wilcoxon sign-rank tests). When compared using the adjusted CDE metric, the difference between receivers remained significant (*p* < 0.05). The 50 m depth receiver had a median adjusted CDE of 1.00 while the receiver at 212 m depth had an adjusted CDE of 0.214 (Fig. 9).

Median RC values for each receiver were not significantly different, with a median value of 0 for the receiver at 50 m depth (no detections were rejected) and a median value of 0.0138 for the receiver at 212 m depth (*p* > 0.05). When adjusted as described above, the difference was significant (*p* < 0.05). The median daily adjusted RC was 0 for the receiver at 50 m depth and 0.110 for the receiver at 212 m. These daily results, which make no assumptions about the number of transmissions sent during the study period, are similarly consistent with our hourly analyses and the mechanistic model’s predictions, supporting the use of our adjusted metrics when CPDI effects are present.

### Experiment 4: depth and distance model validation experiment: 25 May–30 May 2015

Consistent with the mechanistic model’s predictions, the receiver 60 m from the tags detected fewer transmissions than the receiver 508 m from the tags. Shapiro–Wilks’ testing indicated that the distribution of hourly detections were non-normal (*p* < 0.01 and *P* < 0.001 for the receivers at 60 m and 508 m from the tags, respectively). A Wilcoxon sign-rank test used to compare hourly detection counts between the receivers found that the receiver at 508 m recorded significantly more detections per hour than the receiver at 60 m, logging on average over 1.5 more detections per hour (7.67 transmissions per hour compared to 4.88) than its shallow water counterpart (*p* < 0.001), despite the greater distance. The receiver at 508 m range outperformed the receiver at 60 m range in 120 of the 133 h intervals and recorded the same number of transmissions during 4 of the 133 hour intervals. In the nine remaining cases, the receiver at 60 m detected more transmissions than the receiver at 508 m. Although the specific explanation for these nine cases is unknown, it is possible that it was due to fluctuating NLs.

In support of the hypothesis that fewer transmissions detected by the receiver closest to the tag were caused by invalidated ping trains, meta-logs showed that the receiver located 60 m from the tags recorded more individual pings than the receiver at 508 m over the duration of the study (11,277 pings compared to 9,731 pings). Despite this, the 60 m range receiver logged fewer detections of completed transmissions during the same period (674 detections compared to 1,050). These results compare favorably to the mechanistic model, which predicted a CPDI range of 276.6 m.

### Experiment 5: multipath confirmation: 13 July 2016

Of the 900 simulated tag transmissions, only 20 measured outcomes differed from CPDI predictions. Of these, there were four detections where transmissions included simulated multipaths predicted to interfere with detection. The remaining 16 discrepancies occurred when the model predicted detection but no detection was logged by the receiver.

The binomial GLM compared detection or nondetection of a transmission logged by the VR2-W during tank testing to predictions of the CPDI model. Initially, the GLM was fit with predictive CPDI classification, event ID, and their interaction as independent variables. The interaction term was found to be statistically insignificant (*p* > 0.05) so the GLM was refit with just predictive classification and event ID variables (Table 3). In addition to the intercept term, representing the control prediction while simulating the receiver closest to the seafloor during the deep water ranging experiment (experiment 1), two model terms were significant. The most significant term was the predictive classification “with multipath, CPDI predicted” (*p* < 0.001). There was no statistical difference in the number of detections between the control group and the “with multipath, no CPDI predicted” group. These results indicated that the detection of transmissions with simulated multipaths where no CPDI was predicted did not differ from the control group without multipaths, for which detection was also predicted. Conversely, there were significantly fewer detections when the arrival times of simulated multipaths predicted CPDI conditions. Of the factor levels for the event ID model terms, only the condition corresponding to results of the 212 m water depth scenario from the depth-dependent model validation experiment (experiment 3) were significant (*p* < 0.001). Overall, the model explained approximately 81.5% of the observed variance (pseudo *R*^2^ = 0.815) with 81.8% of that total explained variance coming from our predictive CPDI classification.

**Table 3:** Experiment 5 GLM results.

Model term	Estimate	Standard error	*z* Value	Pr (>|*z*|)
Intercept (control/deep water ranging: receiver 1 m)	4.159	0.634	6.559	0
Multipath CPDI predicted	−8.319	0.712	−11.676	0
Multipath no CPDI predicted	1.447	0.803	1.802	0.072
Exp analogue—deep water ranging: receiver 30 m	0	0.742	0	1
Exp analogue—shallow water ranging: receiver 1 m	−1.883	0.766	−2.458	0.014
Exp analogue—shallow water ranging: receiver 7.5 m	15.871	1575.438	0.01	0.992
Exp analogue—shallow water ranging: receiver 15 m	−0.987	0.879	−1.123	0.261
Exp analogue—depth-dependent validation: depth 50 m	−23.299	2746.956	−0.008	0.993
Exp analogue—depth-dependent validation: depth 212 m	−4.565	0.905	−5.047	0
Exp analogue—depth and distance validation: depth 60 m	0	1.891	0	1
Exp analogue—depth and distance validation: depth 508 m	0	1.891	0	1

**Notes:**

Summarized results for the controlled tank experiments fit with a binomial GLM comparing observed detections to outcomes predicted by our CPDI criterion.

Residual deviance: 109.28 on 449 degrees of freedom.

Null deviance: 583.73 on 459 degrees of freedom.

## Discussion

Predicting conditions under which CPDI may occur is important for optimal implementation of acoustic networks and interpretation of study results. The present study demonstrates that relative positions (in both depth and distance) of a receiver and tag can lead to conditions where acoustic energy reflected from the surface and/or seafloor may interfere with detection of the transmission’s ping train. Implementation of a ray tracing mechanistic CPDI model was able to predict when this interference occurred in multiple experiments with a high degree of accuracy.

It has been noted that CPDI may be present in environments particularly amenable to acoustic reflection (Kessel et al., 2015). This stands to reason as TLs incurred during reflection in these environments are low, producing multipaths that are relatively loud. However, particularly for receivers deployed in deep water settings, surface reflections may be enough to produce observable CDPI effects regardless of the reflective properties of the seafloor. Compared to their shallower receiver counterparts, for deeper receivers, reflected acoustic energy has the potential to arrive following the end of the blanking interval with fewer reflections off the surface and/or seafloor. These signals incur fewer TLs due to scattering and reflection than signal energy reflected multiple times. In relatively low noise environments also prone to acoustic reflections, multipath acoustic energy reflected off the surface, seafloor, or some combination of each, may also arrive with sufficient intensity for detection by the receiver, invalidating transmission’s detection, and exacerbating the problem of detection under CPDI conditions.

During our deep and shallow ranging experiments (experiments 1 and 2), some variability in the presence and observed magnitude of CPDI effects can likely be attributed to the number of high output tags used and their variable transmission intervals. The maximum transmissions detected by a receiver of a single tag was 40 of 60 expected hourly transmissions. We believe this was partially a result of the large number of tags used during each ranging experiment (12 in the dep water experiment and 18 in the shallow water experiment), with relatively short transmission intervals (averaging 60 s) resulting in failure to detect transmissions during periods where 2 or more transmissions occurred simultaneously, reducing the overall number of transmissions detected each hour.

Selecting an appropriate transmission interval and power output of study tags is often a tradeoff. The tags used in experiments 1–3 were selected for use in a deep water snapper study with receivers positioned so their detection ranges would overlap in fence/gate configurations. A relatively short transmission interval was selected so multiple transmissions would be emitted by tagged fish swimming between receivers, improving the probability of detecting the presence of an individual. For similar reasons, tags were also high output. Selecting high output tags allowed us to maximize the distance from a receiver that transmissions could be detected and construct a fence from a minimum number of receivers. However, increasing the output level of a tag also increased the received signal level of transmission multipaths which, under sufficient conditions, produce CPDI.

Some hourly variation in the number of total transmissions sent by each tag was expected and may have contributed further variability to the observed hourly data. However, it is unlikely the variable transmission interval accounts for the magnitude of observed CPDI effects as each transmitter has the same variability in transmission interval; thus all tags were expected to have a similar number of hourly transmissions.

Standardizing test results of the depth-dependent model validation experiment using data from receiver meta-logs allowed us to control for discrepancies in variable transmission intervals. The number of synchronization intervals and pings detected are likely underestimates of the true values due to the receiver’s inability to detect transmissions during blanking intervals (Simpfendorfer, Heupel & Collins, 2008). Both synchronization interval and ping data were used to compare between the two depth conditions in the depth-dependent model validation experiment (experiment 3). These may have led to underestimation of the number of transmissions undetected at the deeper receiver but we do not think this had an effect on the overall outcome of the experiment. Relative to the receiver at 50 m water depth, the receiver at 212 m depth showed the effects of CPDI while having comparatively higher daily values for both synchronization intervals detected (3,658 median daily synchronization intervals compared to 1355.5 median daily synchronization intervals) and daily pings detected (11777.5 median daily pings compared to 10844.5 median daily pings). Despite greater detection of individual syncs and pings, this receiver logged 1,039 fewer transmissions per day on average (316.5 median daily detections compared to 1355.5 mean daily detections). This indicates that the deeper receiver detected more individual pings but failed to detect the transmissions. This observation is consistent with transmissions being affected by CPDI. The remaining experiments were not subject to these concerns as each used a design in which all transmissions were detectable by each receiver.

Environmental and anthropogenic factors have been implicated as external sources of variability affecting receiver detection performance (Cagua, 2012; Cagua, Berumen & Tyler, 2013; Gjelland & Hedger, 2013; Mathies et al., 2014). While our mechanistic model does not directly account for background NL, in practice, increased background noise leads to a reduction of the AMDR term and decreases CPDI range. Similarly, lower background NLs may increase both AMDR and CPDI range. Thus, background NLs are accounted for indirectly in the model through the AMDR term. Parameters for the AMDR used in the mechanistic CPDI model were estimated from ranging results by the fit of the candidate GAMs. During periods of increased background noise within the receiver’s detection frequency bandwidth, greater acoustic energy is required to get a signal-to-noise ratio greater than the DT. We suspect that this accounts for the large discrepancy between the AMDR values for the deep water ranging experiment (experiment 1) which took place in deeper and presumably quieter waters than the shallow water ranging experiment (experiment 2) where equipment was positioned near a patchy coral reef and harbor entrance.

Low background NLs in the tank and artificially high signal levels for simulated multipath arrivals produced CPDI at simulated distances far surpassing those observed during shallow and deep water ranging experiments. There was a higher number of detected transmissions in the tank environment for simulated tag transmissions which mimicked distances between tag and receiver where a low number of detections were observed in field experiments and for which CPDI was not expected. Low background NLs in the tank environment meant that signals of weaker intensity were detected by the receiver. In several instances, CPDI was not observed in field results, but was present in the tank experiment analogue. Multipath arrivals in the ocean undergo additional attenuation when reflecting off the sea surface and seafloor interfaces. These losses were not accounted for in the calculation for reducing signal intensity of multipath arrivals in tank simulations. When coupled with the tank’s favorable low noise conditions, we would expect more simulated multipath arrivals to arrive at the receiver with sufficient intensity for detection to produce CPDI in the tank than in the field.

There are a number of study designs and analysis methods that would benefit from the consideration of CPDI. When paired with knowledge of an organism’s swimming speed, this model can be helpful in the selection of an appropriate interval for tag transmissions. An ideal transmission interval will ensure tagged individuals traveling through the detection range of the receiver have a likelihood of detection equal to or greater than some acceptable probability. This is particularly relevant to passive acoustic network arrays where the detection footprints of receivers overlap, such as full coverage, gate, and curtain designs (Heupel, Semmens & Hobday, 2006; Hobday & Pincock, 2011). When applied post-hoc, the mechanistic model for predicting CPDI described here can give some indication of overall network performance and estimate the permeability of overlapping receiver detection footprints. In studies using depth sensor tags to investigate the depth distribution of an organism, detection logs may under-represent depths where CPDI conditions are prevalent, given that the incidence of CPDI is sensitive to tag depth. Studies where receivers are attached to dynamic platforms such as vessels, gliders, autonomous underwater vehicles, and marine animals, should also consider the effect that changes in receiver position and environment depth can have on CPDI and transmission detection. It is also important to understand a receiver’s susceptibility to CPDI when choosing to analyze telemetry data using space state models. In their current implementations to marine animal telemetry, these models rely on both detection and nondetection probabilities to estimate the distance of tagged individuals from a receiver (Pedersen & Weng, 2013; Alós et al., 2016). CPDI may confound position estimates if not accounted for as equivalent detection probabilities can occur at multiple distances from the receiver. Paired with appropriate range testing and knowledge of the study organism’s habitat preferences, the model for CPDI proposed in this study can be used to suggest optimal vertical receiver positioning within the water column. If preferred depth of the study species is unknown, the model can be run over the full depth range or a subset of ranges with only a small increase in computational runtime.

## Conclusion

Close proximity detection interference results in the failure to detect tag transmissions when reflected acoustic energy arrives at a receiver with intensity and timing sufficient to be mistaken for a unique signal. Our results show that when CPDI conditions are present, the shape of a receiver’s detection function includes an area of low detection probability near the receiver. Conditions leading to CPDI can be reasonably predicted by incorporating knowledge of the study environment and a receiver’s detection parameters. Depth is also a key factor in the occurrence of CPDI. Assuming a constant sound speed of 1,530 m/s, CPDI may occur when relative path lengths exceed 400 m. In this example scenario, CPDI arising from the first surface reflection occurs for receivers at depths greater than 200 m. In cases where reflection off both the surface and seafloor are important, the receiver depth for which CPDI occurs will decrease relative to this surface-reflection only case. Relatively quiet and/or highly reflective environments (e.g., hard bottoms) lead to higher signal-to-noise ratios which result in a greater number of multipath arrivals that can be detected at the receiver. These signals potentially interfere with transmission detection, increase the CPDI range, and result in fewer (or potentially no) detections from tagged individuals near receivers.

Modeling for CPDI, therefore, is an important step for designing and interpreting acoustic tagging studies, particularly when working at greater depths. This is particularly a concern as acoustic tracking studies occurring in deeper waters become more common (Starr, Heine & Johnson, 2000; Afonso et al., 2012, 2014; Weng, 2013; Comfort & Weng, 2014; Gray, 2016). Prior to deployment of acoustic hardware, CPDI modeling over known depth distributions, consistent with a study species, can recommend deployment configurations to potentially mitigate CPDI effects. When the depth distribution for a species of interest is unknown, or a receiver network is being used to monitor multiple species with differing depth distributions, modeling over the entire water column can still provide researchers with valuable suggestions for deployment depth with little extra computation time.

## Supplemental Information

10.7717/peerj.4249/supp-1 Supplemental Information 1 Raw data. Raw data used for analysis of components 1, 3, and 4.

10.7717/peerj.4249/supp-2 Supplemental Information 2 Mechanistic Model Implementation–Matlab. A zip file containing the Matlab implementation of the mechanistic model for predicting CPDI and a README file instructing on its use.

10.7717/peerj.4249/supp-3 Supplemental Information 3 Analysis script. Code for performing analysis and generating figures presented in manuscript.

10.7717/peerj.4249/supp-4 Supplemental Information 4 Mechanistic Model Implementation–R. A zip file containing the R implementation of the mechanistic model for predicting CPDI and a README file instructing on its use.

## Additional Information and Declarations

### Data Availability

The following information was supplied regarding data availability: GitHub: https://github.com/stevescherrer/CPDI-Submission-Repository.git.
