# Supplementary material for: Depth- and range-dependent variation in the performance of aquatic telemetry systems: understanding and predicting the susceptibility of acoustic tag–receiver pairs to close proximity detection interference
Source: PeerJ. 2018 Jan 12;6:e4249. doi: 10.7717/peerj.4249 (PMC5768168; doi:10.7717/peerj.4249)
Supplement: Supplemental Information 2 — A zip file containing the Matlab implementation of the mechanistic model for predicting CPDI and a README file instructing on its use. [file peerj-06-4249-s002.zip › Mechanistic Model Implementation - MATLAB/README - CPDI model in Matlab.rtf]

Welcome to the README file for our MATLAB implementation of the Mechanistic Model for Predicting CPDIThe source code for this project can be found at the following GitHub repository:https://github.com/stevescherrer/CPDI-Submission-RepositoryThere are two scripts for our MATLAB implementation. 1. testCPDI.m2. Mechanistic CPDI Model.mTestCPDI.m tests the parameter set provided for CPDI.  It relies on MOI_XYZ.m to provide the underlying model. Both functions take the following arguments.—m = a vector of model parameters in the following order.	xSource - Numeric Value. The horizontal position of the source transmitter (tag) in meters	ySource - 0. Is not used in this model, should be set to zero 	zSource - Numeric Value - Corresponds to the tag depth, measured positive downward from surface. Ex: To measure a tag 1m above a seafloor of 300 m depth use 299. In meters	cBartSource - 0. Is not relevant for implementing this model and can be set to zero	cBardOffset - 0. Is not relevant for implementing this model and can be set to zero	xPhone - Numeric value. The horizontal position of the hydrophone (receiver’s grid location x axis)	yPhone - 0.  Is not relevant for implementing this model and should be set to zero	zPhone - Numeric value. Corresponds to the receiver depth, measured positive downward from surface. Ex: To measure a receiver 1m above a seafloor of 300 m depth use 299.		dWater - Numeric value. The depth of the water column	cWater - Numeric value The sound speed in meters/second	cConstant - 0, scaling value used when this propagation model is used for localization. Not important for this modelarrivePath      (binary vector indicating which arrival paths arrival times must be calculated). This is a vector of 20 elements corresponding to the first 20 arrival paths. The direct path is the first element, followed by a single bottom reflection(B). The third element is a single surface reflection(S). The fourth element is reflected twice, first off the bottom and then surface (BS), the fifth is reflected twice, first the surface bottom reflection (SB), The sixth is reflected three times, the bottom surface bottom reflection (BSB) followed by the seventh element reflected three times first surface, then bottom, then surface reflections (SBS)… etc… For example, a water column of 220 m. Tag is positioned 20 m above seafloor (200 m depth) and Receiver is located 25 m above seafloor (195 m depth). Sound speed in water is 1530 m/s. Receiver is said to be at x = 0 with tag a relative distance of 122 m away.ex: m = [122, 0, 200, 0, 0, 0, 0, 195, 220, 1530, 0] If we were to determine CPDI from the direct path and first 4 multi paths we would declare arrivePath as such:ex: arrivePath = [1,1,1,1,1,0,0,0,0,0,0,0,0,0,0,0,0,0,0,0]After loading both function files into our path, we run the function: testCPDI(m, arrivePath)To Run the function over a set of tag depths, put function in a for loop and update zSource with each loop iteration. To determine best receiver placement, nest for loops so inner loop 
