# Supplementary material for: Depth- and range-dependent variation in the performance of aquatic telemetry systems: understanding and predicting the susceptibility of acoustic tag–receiver pairs to close proximity detection interference
Source: PeerJ. 2018 Jan 12;6:e4249. doi: 10.7717/peerj.4249 (PMC5768168; doi:10.7717/peerj.4249)
Supplement: Supplemental Information 4 — A zip file containing the R implementation of the mechanistic model for predicting CPDI and a README file instructing on its use. [file peerj-06-4249-s004.zip › Mechanistic Model Implemented - R/README - Mechanistic Model in R.rtf]

Welcome to the README file for our R implementation of the Mechanistic Model for Predicting CPDIThe source code for this project can be found at the following address:https://github.com/stevescherrer/CPDI-Submission-Repository/blob/master/Code/Mechanistic%20CPDI%20Model.RAs noted in the scripts comment section, there are two main functions for users such as yourself.1. predict_cpdi_interference()2. rank_receiver_depths()We have also included the function test() with example values to test that the script is working properly. 1. predict_cpdi_interferenceThis function takes a number of arguments and produces a matrix with rows corresponding to transmitter depth and columns corresponding to transmitter distance from the receiver. If the argument plot = TRUE, The function takes the following arguments:— bottom_depth - numeric value. Depth in meters of the study site. In this implementation, it is assumed the study site has a uniform bottom depth. — average_max_detection_radius - numeric value. Maximum distance in meters from a receiver that a tag can be detected. — receiver_depth - numeric value. Depth of receiver  in meters relative to the surface. (ie: If receiver is on rope 3 m above seafloor, receiver depth = bottom depth - 3)—  speed_of_sound - numeric value. Speed of sound in enviornment. Assummed to be 1530 m/sec unless specified otherwise—  max_horizontal_dist - numeric value. Maximum distance model should simulate— evaluation_interval - numeric value.  To reduce computational speed, model can bin search area. By default, model makes a prediction for every 1 meter.  — blanking_interval - numeric value. The receiver's blanking interval in seconds (default is 0.260)— plot - TRUE or FALSE. Whether or not to produce a plot showing where detections are predicted to occur. Light grey indicates positions where a tag can be heard while dark grey are positions where tag is not heardsave_file - TRUE or FALSE. If plot argument is true and save_file argument is true, plot will be saved to working directory.— ...  - Additonal plot options found in plot heat map function. Notably, save_file = TRUE will save a plot to the working directory 2. rank_receiver_depths()This function takes a number of arguments and generates a list of the optimum heights for placing a receiver based on the number of total  positions that a tag can be detectedInputs: — bottom_depth - numeric value. Depth in meters of study site.  — max_horizontal_dist - numeric value . Maximum distance from the receiver model should simulate — ave_max_detection_radius - numeric value. Maximum distance in meters from a receiver that a tag can be detected. — evaluation_interval - numeric value.  To reduce computational speed, model can bin search area. By default, model makes a prediction for every 1 meter.   —  speed_of_sound - numeric value. Speed of sound in enviornment. Assummed to be 1530 m/sec unless specified otherwise— blanking_interval - numeric value. The receiver's blanking interval in seconds (default is 0.260)— tag_depth_range - An optional vector of depths a tag may appear of the format min_depth:max_depth or c(min_depth, max_depth). If a fish is known to be present at depths of 120-300 m, it makes no sense to evaluate positions shallower than 120 m.  
